# Extracting Family History Information From Electronic Health Records: Natural Language Processing Analysis

**DOI:** 10.2196/24020

**Published:** 2021-04-30

**Authors:** Maciej Rybinski, Xiang Dai, Sonit Singh, Sarvnaz Karimi, Anthony Nguyen

**Affiliations:** 1 Commonwealth Scientific and Industrial Research Organisation Sydney Australia; 2 University of Sydney Sydney Australia; 3 Macquarie University Sydney Australia; 4 Commonwealth Scientific and Industrial Research Organisation Brisbane Australia

**Keywords:** information extraction, natural language processing, clinical natural language processing, named entity recognition, sequence tagging, neural language modeling, data augmentation

## Abstract

**Background:**

The prognosis, diagnosis, and treatment of many genetic disorders and familial diseases significantly improve if the family history (FH) of a patient is known. Such information is often written in the free text of clinical notes.

**Objective:**

The aim of this study is to develop automated methods that enable access to FH data through natural language processing.

**Methods:**

We performed information extraction by using transformers to extract disease mentions from notes. We also experimented with rule-based methods for extracting family member (FM) information from text and coreference resolution techniques. We evaluated different transfer learning strategies to improve the annotation of diseases. We provided a thorough error analysis of the contributing factors that affect such information extraction systems.

**Results:**

Our experiments showed that the combination of domain-adaptive pretraining and intermediate-task pretraining achieved an F1 score of 81.63% for the extraction of diseases and FMs from notes when it was tested on a public shared task data set from the National Natural Language Processing Clinical Challenges (N2C2), providing a statistically significant improvement over the baseline (*P*<.001). In comparison, in the 2019 N2C2/Open Health Natural Language Processing Shared Task, the median F1 score of all 17 participating teams was 76.59%.

**Conclusions:**

Our approach, which leverages a state-of-the-art named entity recognition model for disease mention detection coupled with a hybrid method for FM mention detection, achieved an effectiveness that was close to that of the top 3 systems participating in the 2019 N2C2 FH extraction challenge, with only the top system convincingly outperforming our approach in terms of precision.

## Introduction

### Motivation and Contributions

The widespread use of electronic health records (EHRs) is believed to be one of the key enabling factors leading to the improvement of patient outcomes through data analytics. The analysis of EHRs has been successfully carried out for more than a decade in various health care scenarios [1,2]. Nonetheless, a significant proportion of the information stored in digital patient files is trapped in free-text representations. In particular, family history (FH) reports, vital in the diagnosis and treatment of genetic disorders and familial diseases, such as cardiovascular diseases and cancers, are often stored within EHRs as lengthy textual fields.

In the natural language processing (NLP) subfield of artificial intelligence, information extraction (IE) from free text has been studied for decades. However, IE for biomedical and clinical text is one of the most difficult scenarios for 3 main reasons: (1) entities are complex and diverse [3], (2) clinical text is fragmented and contains shorthand terms, and (3) annotated data are scarce.

We describe a system for extracting information contained in FH reports. The aim of our system is to detect family member mentions (family member [FM] type) and detect mentions of diseases (Observation type). It is developed and evaluated within Track 2 of 2019 N2C2/Open Health Natural Language Processing Shared Task [4], subtask 1.

We leverage pretrained biomedical neural language models (LMs) and combine them with rule-based heuristics and coreference resolution to identify diseases (observations) and FMs in clinical notes. Our main contributions are as follows:

- An entity detection system for FH notes with a state-of-the-art named ennity recognition (NER) model for disease mention detection and a set of heuristics for annotation and normalization of FM mentions
- A detailed evaluation of different transfer learning strategies to improve the annotation of diseases
- A discussion of contributions of individual system components in FM mention detection paired with a detailed error analysis
- An analysis of applicability of coreference resolution to the problem of FM annotation

Our experimental evaluation shows that our system performs better than the median for all systems participating in the shared task by a considerable margin. We believe that an architecture such as ours, which uses domain-specific rules where training data are noisy or scarce, has high applicability in the creation of refined training data sets for FH IE.

### Background and Previous Work

#### EHRs - Context

EHRs, the majority of which contain free text, such as clinical notes, discharge summaries, and pathology reports, have led to an improvement in health care quality by electronically documenting patients' medical conditions [5,6]. EHRs are used for various primary and secondary purposes, such as care process modeling, clinical decision support, biomedical research, and epidemiological monitoring of the nation's health. Although NLP and machine learning (ML) applications in clinical text are receiving attention, the progress is limited because of the lack of shared data sets and tools because of privacy and data confidentiality constraints. To overcome these challenges, efforts have been made by shared task organizers, such as the National Natural Language Processing Clinical Challenges (N2C2), to promote clinical NLP research and provide a standard benchmark to evaluate the performance of the proposed systems.

In next subsections, we introduce some of the related IE techniques and provide a summary of past studies on FH extraction.

#### Clinical IE

IE is the process of translating free text into structured data. It often includes 2 tasks: (1) NER, where mentions of named entities are identified in free text, and (2) relations between these named entities are identified. In the clinical setting, these entities can be symptoms, drugs, or diseases [6].

Earlier IE systems often relied on expert rules to identify mentions of predefined entities. Rule-based toolkits specialized for clinical text, such as MetaMap [7,8], rely on external knowledge sources of biomedical terms, such as the SPECIALIST lexicon, and use complex rules to identify all possible mention variants of an entity, including acronyms, abbreviations, synonyms, or derivational variants. These tools can usually achieve high precision (when the identified mentions are indeed correct) at the expense of low recall (when many mentions are missed). Another shortcoming of rule-based systems is that expensive human efforts are required to maintain the resources and to expand the rules, enabling them to stay up to date with evolving language use and domain knowledge.

ML-based systems [9,10] replace *hard* rules as *soft* features and estimate the importance (weights) of features using annotated training data. Despite the successful applications of ML-based IE systems, they still display domain discrepancies. That is, the distribution of training data, based on which feature templates are designed and weights are estimated, differs from the data distribution where the system is employed. Therefore, the quality of manually designed feature templates is critical for the system. These features should be informative and should generalize for unseen data.

To alleviate the burden of manually building feature templates, deep learning models have been increasingly applied on clinical IE tasks. A key idea that enables the success of recent deep learning–based models in NLP is that word meanings can be encoded in dense vectors via pretraining on raw text [11-13]. Efforts along this direction in clinical NLP focus on obtaining better word representations for clinical text [14]. For example, Alsentzer et al [15] and Huang et al [16] pretrained Bidirectional Encoder Representations from Transformers (BERT) models on clinical notes and achieved better performance than BERTs pretrained on generic-domain text. Zhang et al [17] investigated strategies to adapt generic-domain embeddings to the clinical domain. Another direction in clinical IE is to identify complex entities that are less common in generic NLP. For example, Wang and Lu [18] and Dai et al [3] proposed models to recognize overlapping or discontinuous entities that usually represent compositional concepts that differ from concepts represented by individual components.

#### Previous Work

FH plays an important role in the decision-making process of diagnosis and treatment of medical conditions, as it captures shared genetic variations among FMs. Information such as age, gender, and the degree of relatives are also considered in the risk assignment of various common diseases [19]. Many care process models use FH information for decision making in diagnosis and treatment [5]. Modern health care systems usually record FH through structured forms, including free-text sections, which are filled either by a patient or by a clinician. Polubriaginof et al [20] assessed the quality of the FH captured in EHRs. They found that free-text observations were more comprehensive than structured observations, which motivated our study.

The task of extracting FH from clinical notes is challenging because the information can be spread in the patient's progress notes [21]. In addition, FH information is expressed via relations between named entities and may contain contextual information such as certainty and negation, vital statistics, and age modifiers [22]. If we predict that a patient is at an increased risk of developing a certain disease based on FH, we could potentially diagnose it early, leading to early treatment. Computer-based tools can facilitate the effective use of FH and, therefore, provide better personalized care [20]. To provide comprehensive patient-provided FH data to physicians, there is a need for NLP systems that can extract FH from text. The task of FH IE generally includes NER or relation extraction [23].

Friedlin and McDonald [24] developed a rule-based system, a Regenstrief Data Extraction (REX) tool, for extracting and coding FH data from hospital admission notes. The REX tool first locates and extracts the FH section from the admission notes. It then attempts to identify diseases. This system led to a sensitivity of 93% and a positive predictive value of 97% on the 1 years’ worth of hospital admission notes. However, the study was limited to only 12 diseases. Goryachev et al [25] developed a rule-based system to identify and extract FH from discharge summaries and outpatient clinical notes. The Health Information Text Extraction [26], which is built on top of a General Architecture for Text Engineering [27] framework, is used to parse discharge summaries and patient notes. Experiments on a set of 2000 reports yielded 85% precision and 87% recall. The architecture yielded promising results; however, the validation set used in the study was small.

Lewis et al [21] followed a 2-step method that selects candidate FH sentences based on the presence of words such as *mother* or *brother* and then uses a set of dependency-based syntactic patterns to extract appropriate diagnoses and identify the FMs referred to. This study restricted observations to concepts that could be mapped to the International Classification of Diseases, ninth edition, codes. They also limited their work to per-sentence IE without considering any cross-sentence anaphoric or coreference resolution. In our study, we experimented with a coreference resolution and evaluated it in our setup.

Almeida and Matos [28] developed rule-based methods using dependency parsing and a phrase-characteristic extraction approach to extract FH information from clinical notes. They used Stanford CoreNLP [29] to process the data, perform dependency parsing and coreference resolution, and then annotate their data for all FMs and observations. This way, context from previous sentences was also considered. On the N2C2 2019 shared task, which is the same data set that we used, they reported F1 scores of 72% and 74% for the discovery of FMs and observations, respectively. Their approach relied on heuristics to detect arguments of relations, such as using a predefined list of family relationships and diseases or making use of it as arguments in the noun phrases that are detected close to the suspected relationship markers. However, finding relation arguments is challenging because of their variable lengths.

When NER and relation extraction are applied in a pipeline, the error propagates from the NER module to the relation extraction module. To avoid this error propagation, Shi et al [23] proposed a joint learning method that tackles both of these tasks by sharing parameters in a unified neural network framework. The FH IE is performed at different levels, including FMs, observation, and living status and the side of the family (maternal, paternal, and not available). Each input token is represented by word embeddings and corresponding Part-of-Speech embeddings and is given as an input to the bidirectional long short-term memory (BiLSTM) model. Their proposed model ranked first in the 2018 N2C2 FH extraction challenge. They achieved an F1 score of 89% for entity identification and 64% for FH extraction. On the basis of the error analysis, the authors found that a large number of errors are caused by indirect relatives, which can be improved by considering relations among relatives, a feature we incorporate in this study.

Dai [30] formulated the FH IE task as a sequence-labeling problem in which a neural sequence-labeling model was employed along with different tag schemes to distinguish FMs and their observations. They proposed a BiLSTM-Conditional Random Fields (CRFs) model with 3 layers: the character sequence representation layer, the word sequence representation layer, and the inference layer. The proposed method achieved an F1 score of approximately 85% on the test set, which ranked second in the FH entity recognition subtask of the 2018 N2C2 FH extraction challenge. Although the proposed BiLSTM-CRF network is effective in modeling contextual information and label dependencies, it has limitations in that the network can only exploit contexts within individual sequences (sentences) but cannot obtain context from cross-sentence information. To overcome this limitation, Dai et al [31] introduced a neural attention model to exploit cross-sentence information to identify mentions.

Zhan et al [32] fine-tuned the BERT model by including an additional biaffine classifier adapted from the dependency parsing to extract FH mentions.

#### FH Extraction Task

FH IE, as defined in the N2C2 FH 2019 shared task, includes the following 2 subtasks:

1. Entity identification, including FMs, the side of family (paternal, maternal, and not applicable [NA]), and observation (disease)
2. Relations between FMs, including observations (negated or not) and their living status.

The possible FMs in this task are father, mother, parent, sister, brother, daughter, son, child, grandmother, grandfather, grandparent, cousin, sibling, aunt, and uncle. Other relatives, such as spouses (not blood related), nieces, and nephews were excluded. For first-degree relatives—parents, children, and siblings—the side of family is NA.

In relation extraction, a *living status score* is defined per extracted FM to encode whether they are alive and healthy. In this study, however, we focused only on the entity identification subtask.

#### Data Set

The data set for the FH task was curated from synthetic English patient notes, which were randomly sampled from the Mayo Employee and Community Health cohort. It contains 216 notes, which we refer to as *documents*, from which 99 documents are for training and 117 for testing. A total of 2 annotators and 1 adjudicator annotated the corpus, with an interannotator agreement of 0.84 for entities and 0.70 for relations. The overall statistics of the corpus are shown in Table 1.

**Table 1:** Statistics (counts) of entities and relations in the National Natural Language Processing Clinical Challenges family history data set.

Data set’s artifact	Training size, n	Test size, n
Document	99	117
Family member	803	760
Observations	978	1062

#### Evaluation Metrics

For entity extraction, a system extracts either a triplet *(document identifier, family member,* and *side of family)* for FM mentions or a pair *(document identifier* and *text of observation)* for observation mentions. These triplets and pairs were matched against the gold standard.

Observation partial matches are acceptable. For example, *diabetes* is accepted for *diabetes type 2*. The standard F1 score, precision, and recall metrics are used to evaluate the effectiveness of the proposed models as follows:



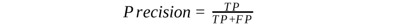





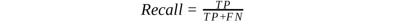





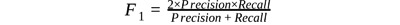



TP denotes true positive, FP denotes false positive, and FN denotes false negative.

Importantly, recall and precision are defined on sets of annotations pertinent to each document. That is, a document can mention *cancer* multiple times, but detection of any of these mentions contributes to the TP count only once. Conversely, the lack of detection of any of these mentions contributes only once to the FN count.

For statistical significance testing, we use a paired approximate randomization test [33] for pairwise comparisons between system variants. We obtain the significance levels by running 9999 pseudorandomized shuffles of the test set results.

## Methods

### Overview

**Figure 1:**
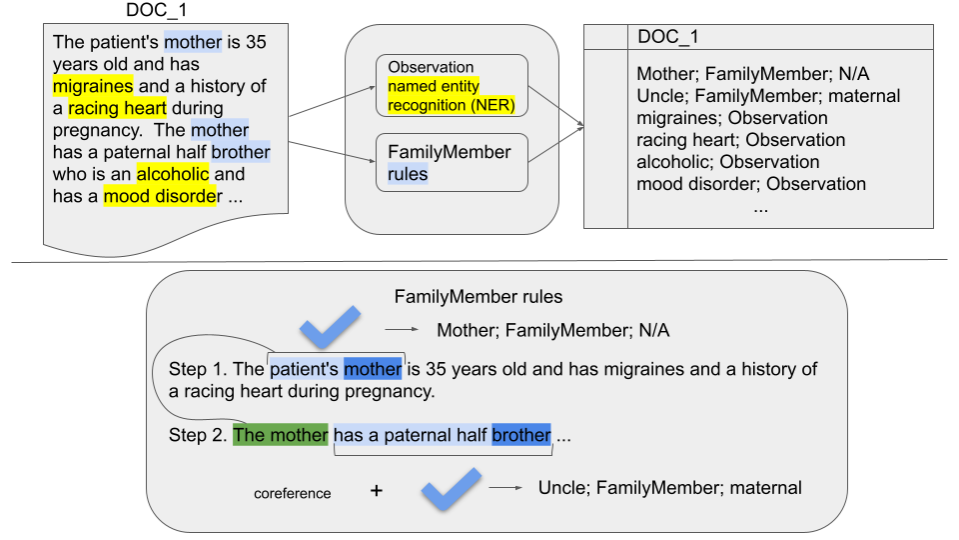
Overview of the system and the FamilyMember mention detection. N/A: not applicable; NER: named entity recognition.

Our task consists of detecting 2 types of mentions: Observation and FamilyMember. The dual objective of the task is reflected in the design of our system, in which the disease mentions are detected with an ML-based NER component, whereas FamilyMember mentions are detected with a hybrid (rule based, with some ML components) module. The overall architecture, together with the inputs and outputs, is illustrated in the top part of Figure 1.

### Observation-NER

#### Problem formulation

We formulated the observation-NER as a sentence-level sequence tagging problem, in which each word in the sentence is assigned a tag. The tag, which uses the Beginning-Inside-Outside schema [34], can be used to infer whether the word is the first word within a mention or inside a mention or does not belong to any mention.

The sequence tagger we use is a state-of-the-art model: the BERT-CRF model [11,35]. It takes advantage of large-scale pretrained LMs using BERT to create contextual representations for each word and a probabilistic graphical model using CRFs [36] to capture dependencies between neighboring tags.

#### BERT-Based Encoder

Given a sentence, the tokenizer, coupled with the pretrained BERT model, first converts each word in the sentence into word pieces. That is, if the original word does not exist in the vocabulary of the tokenizer, it will be segmented into several units from the vocabulary [37]. Then, the word pieces are mapped to dense vectors via a lookup table (also known as token embeddings). Finally, the sum of token embeddings and positional embeddings, which indicate the position of each token in the sequence, is fed into a stack of multihead self-attention and fully connected feed-forward layers [38]. Following the work by Devlin et al [39], we use the final outputs corresponding to the first word piece within each word as the word representation.

#### CRFs in NER

Instead of assigning a tag to each word independently, we modeled them jointly using CRFs [40]. That is, given a sequence of word representations *X= (x_1_, x_2_,..., x_n_)*, we aim to predict a sequence of tags (*Y= (y_1_, y_2_,..., y_n_)*) that has the maximum probability over all possible tag sequences. This conditional probability can be calculated using the following equations:



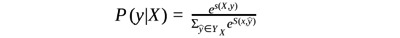





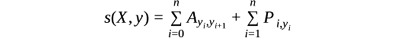



*A_i,j_* is the compatibility score of a transition from the tag *i* to tag *j* and *P_i,j_* is the score of the tag j given *X_i_*.

The parameters from both BERT and CRFs are trained to maximize the conditional probability of the gold tag sequence, given the training sentences.

#### Enhancing BERT

The vanilla BERT model is pretrained using generic-domain data such as books and Wikipedia, which are very dissimilar to task data. A previous study has shown that the effectiveness of pretrained LMs is highly affected by the similarity between source pretraining and target task data [41].

Thus, we explored 2 approaches to improve the effectiveness of vanilla BERT on the target task: domain-adaptive pretraining (DAPT) [42] and intermediate-task pretraining (ITPT) [43].

##### DAPT Approach

The DAPT approach consists of continued pretraining of BERT on a large volume of unlabeled in-domain text. The training uses a masked language modeling objective to adapt the weights of BERT to the domain of the target task. We use BioBERT [44] and ClinicalBERT [15] as proxies for DAPT. These models employed continued BERT pretraining on biomedical scientific papers and hospital discharge summaries.

##### ITPT Approach

ITPT consists of the pretraining of BERT together with CRFs by training on a target task-related NER data set (usually annotated with similar entity types). The training uses the sequence tagging objective to jointly optimize the weights of both BERT and CRF layers toward the specific task. We used the National Center for Biotechnology Information (NCBI)-disease [11] data set for ITPT. This data set consists of 793 PubMed abstracts that are fully annotated at the mention and concept levels. It contains 6892 disease mentions, which are mapped to 790 unique disease concepts. The motivation for this choice is 2-fold. First, we used the NCBI-disease data set because of the semantic overlap between the *Disease* and *Observation* concepts and because of the size of the NCBI-disease data set, which is larger than the in-domain data (NCBI-disease consists of nearly 800 documents, with almost 7000 disease mentions). Second, this choice results in a more direct comparison with our off-the-shelf baseline, which was trained on the same NCBI-disease corpus (our experimental setup is explained in more detail at the beginning of the *Results* section).

**Figure 2:**
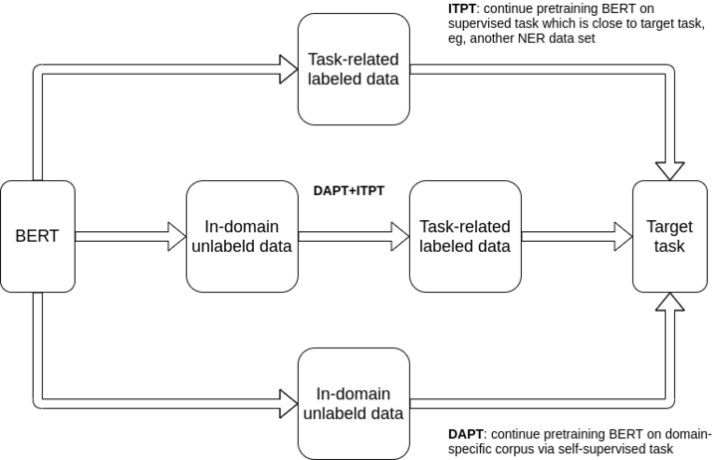
Different approaches to enhance Bidirectional Encoder Representations from Transformers for a given target task (domain-adaptive pretraining and intermediate-task pretraining). BERT: Bidirectional Encoder Representations from Transformers; DAPT: domain-adaptive pretraining; ITPT: intermediate-task pretraining; NER: named entity recognition.

We also explored the combination of these 2 approaches, that is, DAPT and ITPT. A high-level comparison of these 3 approaches is presented in Figure 2.

After DAPT or ITPT, we continued to fine-tune the model weights of the target task's training data.

Owing to the aforementioned semantic overlap between classes of interest, NCBI-disease was our first choice for ITPT. Nonetheless, for the sake of completeness, we also present an ITPT evaluation (for all DAPT configurations) for other publicly available candidate data sets, which involve annotation of diseases, that is, Integrating Biology and the Bedside (i2B2) 2010 [45] and Shared Annotated Resources - Conference and Labs of the Evaluation Forum 2013 [46].

Details of implementation and training of our BERT-CRF models are outlined in Multimedia Appendix 1.

### FamilyMember Mentions

The FamilyMember mentions’ detection often requires an out-of-sentence context to make correct inferences. In the example shown in Figure 1, an out-of-sentence context is needed to resolve coreference to “the mother” and correctly normalize the *brother* or *uncle* mention. Another instance where a broader document context is required is deciding whether the information provided in a given fragment of an FH note pertains to the patient's or their partner's family. The task is focused on extracting the information on the patient's blood relatives; therefore, the mentions of the partner's family should not be annotated, although at the sentence level, the information can be identical.

Given the moderate size of the training corpus of approximately 100 documents and the complexity of the FamilyMember normalization task, which entails multiple entity classes, during our participation in the shared task, we opted for a rule-based approach enhanced with some ML elements. This early design choice determines the scope of our focus; however, we compare our approach with state-of-the-art deep learning baselines trained on the available in-domain data.

In our hybrid system, the documents are analyzed sentence by sentence with a series of pattern-matching rules. The previous sentence is used as context when producing FamilyMember annotations for a given sentence (we split each document into sentence-level bigrams). We experimented with a state-of-the-art coreference resolution model *neuralcoref* [47]. Coreference resolution is used on pairs of consecutive sentences to incorporate context information from possessive pronouns (eg, *her son*) or other third-person pronouns (*she has a son*) and alternative references (eg, *this woman has a son*).

To detect paragraphs of the notes containing information on the partner's family, we incorporate a state-of-the-art text classification model. Owing to the lack of dedicated partner-paragraph annotations, we fine-tune a BERT [39] model on the available training data. We formulate the task as a binary classification problem, where the model predicts whether a given paragraph is valid (containing patient-focused information). The previous paragraph is also fed to the model to provide contextual information. We derive validity from the existing training data. That is, a paragraph is valid if at least one annotation is present within its scope in the training data set. At the annotation time, we skip sentences predicted to be part of invalid paragraphs by the model.

Our first step is to predict the validity of each of the paragraphs of an FH note using a BERT-based paragraph filter. This step results in filtering out the paragraphs that are predicted to be invalid by the model. We then iterate all the remaining document sentences to create sentence-level annotations. These annotations are then put into one document-level annotation set. The procedure for FamilyMember mentions’ detection within the scope of a sentence, given a previous sentence and its annotations as context for coreference, consists of the following steps:

1. Check if a sentence is not part of an invalid paragraph. If it is, we skip to the next sentence.
2. Detect candidate mentions in the second sentence (predictions for FamilyMember for the previous sentence are already available); candidate mentions are occurrences of words denoting family relationships relevant to the task as per the task definition, such as *brother, sister, mother, and father.*
3. Build a graph of candidate-candidate relationships. For example, an expression *mother's sister* would result in vertices *mother* and *sister* and a directed edge from *mother* to *sister*; this graph incorporates coreference information.
4. Generate FamilyMember annotations from the graph structure according to a set of rules. For example, the mother-to-sister structure would generate annotations Mother-NA (not applicable) and Aunt-Maternal.

To build a graph of candidate-candidate relationships, we look for specific linguistic patterns between pairs of adjacent candidate mentions. These patterns are “X's *Y, X*has/had *Y, Y of *X,” where X and Y denote candidates such as *brother*, *sister*, or *uncle* and the * symbol denotes a wildcard matching any text. We also detect candidate-candidate relationships as adjacency of candidate mentions to expressions linked with coreference resolution to other candidates or FamilyMember mentions from the previous sentence. For example, if in the sentence pair “Mr. Williams' mother is alive and well. She has an older sister...,” the word *mother* is annotated with as *Mother* and word *She* falls into the same coreference cluster as the word *mother*, and then a Mother-Sister relationship will be added to the graph as an X*has*Y pattern is triggered. Downstream, this relationship is used to normalize the annotation of *the sister* according to the rules (to Aunt, Maternal).

**Figure 3:**
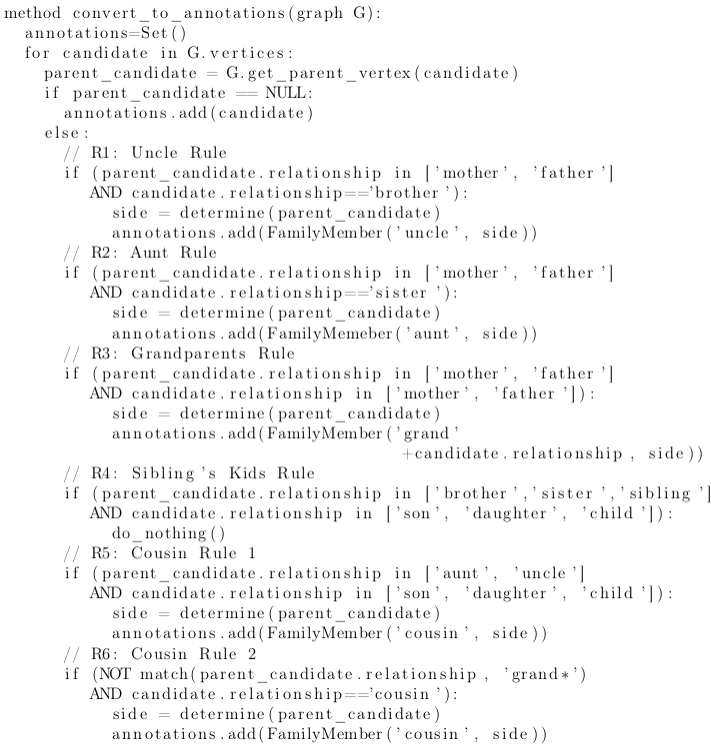
A pseudocode representation of the rule-based processing.

To convert the candidate graph to a final representation, we apply a set of rules to each of the vertices. The procedure, together with these rules, is presented in the pseudocode in Figure 3.

In addition, we apply a simple heuristic approach to determine the family side for those annotations where the side of the family cannot be determined by inspecting the parent node in the candidate graph. We look for the last occurrence in the text of words *maternal*, *mother* or *paternal*, *father*, before the given candidate is mentioned. The maternal or paternal status is determined according to this last occurrence. We only assign *NA* if none of these words appear in the document before the candidate is mentioned.

## Results

### Observation Extraction

The gold standard tags are recreated naively by string matching the gold annotations provided. For example, given a gold annotation *mental retardation,* we find all occurrences of this annotation in the corresponding document and assign *B-Observation I-Observation* tags to all the identified spans. We select the first 18 documents from the training set as the development set. The trained model that is most effective on the development set, measured using the span-level F1 score, is used to evaluate the test set. In addition to the different variants of BERT models, we use an off-the-shelf disorder NER model as the baseline [48].

We present the results of our main experiments with the Observation annotation in Table 2. We achieve the best results for a BERT model using both DAPT and ITPT. We provide a detailed discussion of these results in the *Discussion* section.

The results of the additional ITPT experiments with i2b2 2010 and ShARe-CLEF (Shared Annotated Resources-Conference and Labs of the Evaluation Forum) 2013 are presented in Table 3. The results indicate that, although improvements from ITPT alone are comparable with those obtained with the NCBI-disease data set, the DAPT+ITPT combination with the alternative disease annotation data sets is less successful.

**Table 2:** Evaluation results on Observation concepts in the test set^a^.

Method	Precision		Recall		F1 score	
	Value	*P* value	Value	*P* value	Value	*P* value
Stanza [48]	81.5	N/A^b^	75.0	N/A	78.1	N/A
BERT^c^ (baseline), mean (SD)	70.7 (2.7)	N/A	87.3 (1.5)	N/A	78.1 (1.1)	N/A
DAPT^d^ (BioBERT), mean (SD)	73.4^e^ (2.2)	<.001	86.5 (1.7)	<.001	79.4 (0.6)	.08
DAPT (ClinicalBERT), mean (SD)	76.2^e^ (3.5)	<.001	83.4 (3.1)	<.001	79.5^e^ (1.0)	.002
ITPT^f^ (NCBI^g^-disease), mean (SD)	75.0^e^ (1.8)	<.001	85.3 (1.1)	>.99	79.8^e^ (0.6)	<.001
DAPT (BioBERT)+ITPT (NCBI-disease), mean (SD)	77.7^e^ (2.6)	<.001	85.1 (2.8)	.08	81.1^e^ (1.1)	<.001
DAPT (ClinicalBERT)+ITPT (NCBI-disease), mean (SD)	78.6^e^ (3.2)	<.001	84.4 (1.5)	.56	81.3^e^ (1.2)	<.001

^a^Document-level precision, recall, and F1 score are reported using official evaluation scripts.

^b^N/A: not applicable.

^c^BERT: Bidirectional Encoder Representations from Transformers.

^c^DAPT: domain-adaptive pretraining.

^e^Represents results that are significantly better than the Bidirectional Encoder Representations from Transformers baseline (approximate randomization test; *P*=.05). Although the recall of baseline Bidirectional Encoder Representations from Transformers is the highest, the differences are not significant except those for 2 domain-adaptive pretraining variants.

^f^ITPT: intermediate-task pretraining.

^g^NCBI: National Center for Biotechnology Information.

**Table 3:** Evaluation results on Observation concepts in the test set for different intermediate-task pretraining and domain-adaptive pretraining combinations^a^.

ITPT^b^ and BERT^c^ model		Precision, mean (SD)	Recall, mean (SD)	F1 score, mean (SD)
BERT		75.0 (1.8)	85.3 (1.1)	79.8 (0.6)
**NCBI^d^-disease**				
	+DAPT^e^ (BioBERT)	77.7 (2.6)	85.1 (2.8)	81.1 (1.1)
	+DAPT (ClinicalBERT)	78.6 (3.2)	84.4 (1.5)	81.3 (1.2)
	BERT	71.6 (3.4)	88.9 (2.4)	79.2 (1.5)
**i2b2^f^ 2010**				
	+DAPT (BioBERT)	75.6 (1.9)	86.2 (1.4)	80.5 (1.4)
	+DAPT (ClinicalBERT)	73.2 (2.0)	89.0 (1.8)	80.3 (0.7)
	BERT	70.7 (2.7)	88.7 (1.5)	78.6 (1.3)
**ShARe-CLEF^g^ 2013**				
	+DAPT (BioBERT)	72.9 (2.5)	88.3 (2.3)	79.8 (0.8)
	+DAPT (ClinicalBERT)	74.2 (2.6)	86.5 (3.8)	79.8 (0.9)

^a^Document-level precision, recall, and F1 score are reported using official evaluation scripts.

^b^ITPT: intermediate-task pretraining.

^c^BERT: Bidirectional Encoder Representations from Transformers.

^d^NCBI: National Center for Biotechnology Information.

^e^DAPT: domain-adaptive pretraining.

^f^i2b2: Integrating Biology and the Bedside.

^g^ShARe-CLEF: Shared Annotated Resources-Conference and Labs of the Evaluation Forum.

### FamilyMember Extraction

We experimented with the different settings of our approach by evaluating, both on training and test sets, different combinations of the elements of our systems. In addition, we experimented with removing *child*, *sibling*, *parent*, and *grandparent* from the set of relationships, as we hypothesized that the corresponding words are not often used to introduce a particular FM (eg, “She has 4 siblings: two brothers and two sisters”). We obtained the best results on the test set for a system with a restricted set of relationships, using all the rule 1 (R1) to R6 and with BERT-based paragraph filtering, but without the coreference resolution.

The performance of the best system is presented in the first row of Table 4. Subsequent rows demonstrate the impact of modifying the best run by adding the remaining relations (row 2), adding coreference resolution (row 3), removing the BERT paragraph filter (row 4), and removing rules R1-R6 (rows 5-10). Row 11 shows a baseline system with no rules, no paragraph filter, and no coreference resolution, working with the full set of relations.

We compare the results obtained with our hybrid approach with those obtained with a BERT-CRF baseline, identical to those employed for disease annotation. For the sake of completeness, we include baseline results for domain-adapted flavors of BERT—BioBERT and ClinicalBERT.

**Table 4:** FamilyMember detection for different settings of the system.

Row	Number of relations^a^	Coreference	R1^b^	R2	R3	R4	R5	R6	BPF^c^	Training, precision (*P* value)	Test, precision (*P* value)	Training, recall (*P* value)	Test, recall (*P* value)	Training, F1 score (*P* value)	Test, F1 score (*P* value)
(1)	11	—^d^	✓^e^	✓	✓	✓	✓	✓	✓	90.34^f^	81.38^f^	85.60^f^	82.91	87.91^f^	*82.14^f,g^*
(2)	15	—	✓	✓	✓	✓	✓	✓	✓	86.00^f^^,h^ (<.001)	73.73^f^^,h^ (<.001)	89.35^f^^,h^ (<.001)	*86.67*^g,h^ (<.001)	87.64^f^ (.68)	79.68^f^^,h^ (<.001)
(3)	11	✓	✓	✓	✓	✓	✓	✓	✓	88.07^f^^,h^ (<.001)	77.59^f^^,h^ (<.001)	83.05^f^^,h^ (<.001)	79.78^f^^,h^ (<.001)	85.49^h^ (<.001)	78.67^f^^,h^ (<.001)
(4)	11	—	✓	✓	✓	✓	✓	✓	—	87.42^f^^,h^ (<.001)	77.98^f^^,h^ (<.001)	86.50^f^^,h^ (.03)	83.85 (.13)	86.96^f^^,h^ (.04)	80.81^f^^,h^ (.01)
(5)	11	—	—	✓	✓	✓	✓	✓	✓	90.04^f^ (.51)	81.61^f^ (.40)	85.45^f^ (>.99)	82.13 (.07)	87.69^f^ (.51)	81.87^f^ (.35)
(6)	11	—	✓	—	✓	✓	✓	✓	✓	90.06^f^ (.51)	*81.71*^f,g^ (.14)	85.60^f^ (>.99)	81.97^h^ (.03)	87.77^f^ (.51)	81.84^f^ (.19)
(7)	11	—	✓	✓	—	✓	✓	✓	✓	*90.64*^f,g^ (.50)	81.60^f^ (.59)	85.75^f^ (>.99)	81.34^f^ (.09)	*88.13*^f,g^ (.50)	81.47^f^ (.25)
(8)	11	—	✓	✓	✓	—	✓	✓	✓	89.79^f^ (.14)	80.15^f^^,h^ (.004)	85.75^f^ (>.99)	83.54 (.13)	87.73^f^ (.26)	81.81^f^ (.27)
(9)	11	—	✓	✓	✓	—	—	✓	✓	90.62^f^ (.73)	81.13^f^ (.56)	85.45^f^ (>.99)	82.91 (>.99)	87.96^f^ (>.99)	82.01^f^ (.74)
(10)	11	—	✓	✓	✓	✓	✓	—	✓	90.34^f^ (>.99)	81.38^f^ (>.99)	85.60^f^ (>.99)	82.91 (>.99)	87.91^f^ (>.99)	*82.14*^f,g^ (>.99)
(11)	15	—	—	—	—	—	—	—	—	81.52^h^ (>.001)	69.01^h^ (>.001)	*89.95*^h^ (>.001)	84.48 (.38)	85.53^h^ (.01)	75.96^h^ (>.001)
(12)^i^		—	—	—	—	—	—	—	—	N/A^j^	79.72^f^ (.33)	N/A	81.35 (.45)	N/A	80.35^f^ (.26)
(13)^k^		—	—	—	—	—	—	—	—	N/A	81.55^f^ (.95)	N/A	81.03 (.462)	N/A	81.29^f^ (.70)
(14)^l^		—	—	—	—	—	—	—	—	N/A	82.71^f^ (.62)	N/A	79.47 (.17)	N/A	81.06^f^ (.62)

^a^Denotes size of the relationship set.

^b^R1-R6 denote uncle rule, aunt rule, grandparents rule, sibling's kids rule, cousin rule 1, and cousin rule 2, respectively.

^c^BPF: Bidirectional Encoder Representations from Transformers–based paragraph filter.

^d^Not available.

^e^Denotes that the corresponding rule is applicable.

^f^Denotes statistically significant (*P*≤.05) difference from the baseline (row 11).

^g^We report the *P* values corresponding to the test against the best system. Highest measured value is denoted in italics.

^h^Denotes statistically significant (*P*≤.05) difference from the top system (row 1).

^i^Bidirectional Encoder Representations from Transformer-Conditional Random Field baseline results on the test set for Bidirectional Encoder Representations from Transformer.

^j^N/A: not applicable.

^k^Bidirectional Encoder Representations from Transformer-Conditional Random Field baseline results on the test set for BioBERT.

^l^Bidirectional Encoder Representations from Transformer-Conditional Random Field baseline results on the test set for ClinicalBERT.

## Discussion

### Principal Findings

Challenges of recognizing diseases in clinical narratives, such as a wide variety of naming patterns and data anonymization, have been widely studied in the literature [49,50].

Therefore, we provide only a discussion on disease identification that relates specifically to FH extraction tasks and a detailed discussion on FM identification.

### Annotation of Observations

#### Impact of Domain Adaptation

From Table 2, we observe that both DAPT and ITPT can improve over the baseline of the BERT-CRF model, and combining these 2 approaches first with DAPT and then ITPT achieves the best F1 score. On the basis of this result, we argue that DAPT and ITPT can complement each other. In other words, they enhance pretrained LMs by providing different inductive biases. We hypothesize that in the ideal scenario, DAPT enforces the model to be more compatible with the language distribution of the target data and ITPT enforces the model to pay more attention to features that are informative to the NER task.

The aforementioned hypothesis can also be used to explain the results presented in Table 3. We observe that NER *problem* and *disorder* classes (i2b2 and ShARe-CLEF, respectively) are less semantically aligned to our *Observation* class than *Disease* from NCBI. In particular, a large proportion of the *problem* and *disorder* mentions could be classified as *symptoms* (eg, *headaches*, *fever*, and *pain*). Disease names annotated in NCBI-disease seem closer to our target task's *Observation* entity category. It is possible that the alternative ITPT data sets provide an isolated improvement by exposing the model to documents similar to that of the target corpus but offer little improvement when combined with DAPT (which, we assume, already provides this inductive bias).

#### Error Analysis

To provide some insight into the role of task-specific fine-tuning with BERT-like models, we provide a detailed error analysis performed on the outputs generated by an off-the-shelf baseline (trained on the NCBI-disease data set, not tuned on the FH extraction task) and our best system, which is ClinicalBERT with ITPT on the NCBI-disease data set.

The error analysis, apart from counts of FP and FN errors, involves a fine-grained classification of 50 errors of each type (FN/FP) per model. The errors were sampled by taking the first 50 errors of a given type from the output log with randomly shuffled documents.

We classify FP errors into the following categories:

- True FPs: The span does not cover a valid Observation candidate. For example, “The patient's father had six-vessel bypass surgery at age 56.”
- Relative error: The Observation mention by itself is identified correctly but is linked to a relative who is not a suitable candidate for a FamilyMember annotation (eg, a great-uncle would be an example of a too distant relationship, according to the annotation guidelines provided for the task). Importantly, this class of FP errors also covers disease mentions pertaining to the family of the patient’s partner (thus, not related by blood); for example, “His [husband's] brother died at age 14 of suicide and was thought to have depression.” Note that errors are classified as relative errors if the identified Observation looks correct, and it can be linked to a non-FamilyMember; the annotation is missing from the gold standard test data set; for example, the gold standard annotations expect a span containing stomach cancer, a string that does not appear in the corresponding document.
- Nonobservation errors: FNs where the gold-standard annotation is missed by the system, although it appears in the document, but it could be debated whether it constitutes an actual Observation. For example, “She has some freckles.”
- Questionable and nonerrors: The candidate mention looks correct and is linked to a valid FamilyMember candidate. For example, “Mrs. William's sister has had three miscarriages and a son.”
- Trauma or procedure errors: The predicted span includes a name of a procedure or a traumatic injury. For example, “These last two maternal aunts have had hysterectomies.”
- Negation errors: The predicted span covers a valid Observation candidate, but it appears in a negated and often general context. For example, “There is no known history of ADHD or schizophrenia.”

We propose the following categorization for the FN errors:

- True FNs: An actual valid observation was missed by the model. For example, “Her father is 53 with high cholesterol.”
- Gold standard errors: Errors in the gold standard.
- Mental health/substance abuse–related errors: FNs where the models fail to annotate mental health conditions or addictions. We present this special case of true FNs separately, as the evaluated models particularly struggle with detection of this type of observations. For example, “Maternal grandfather, age 67, smokes but is healthy.”

Overall, the off-the-shelf baseline yielded 166 FPs and 244 FNs, with 733 correct annotations. ClinicalBERT-ITPT generated 150 FPs, 172 FNs, and 805 correctly identified mentions. For Stanza, the off-the-shelf baseline values are shown in Table 2. For ClinicalBERT+ITPT, we analyze the run that achieves the highest F1 score among all 5 experimental runs (0.8333 F1 score, 0.8429 precision, and 0.8240 recall).

Table 5 shows the distribution of the error classes over the evaluated sample of FPs. The distribution of the FN errors is shown in Table 6.

An inspection of FPs reveals that for both models, the main source of error is the annotation of observations pertaining to FMs that are not related by blood to the patient (eg, partner's family) or the family relation is too distant (eg, great-grandparents). The BERT-based model alleviates this problem by fine-tuning, at least to a certain degree. However, as the observation-NER is done on a stand-alone basis (ie, without the joint modeling of Observation and FamilyMember spans), the context awareness of the BERT-based model regarding family relationships remains low.

Both models lead to approximately 20% of FPs that appear to be correct; however, they are not present in the gold standard annotations.

**Table 5:** Results (counts) of error analysis for false-positive errors for the Observation entity type.

Error type	Stanza [48], n	ClinicalBERT^a^ with ITPT^b^, n
Relative	31	20
Nonerror	10	9
Trauma or procedure	2	6
Negation	7	9
True	0	6

^a^BERT: Bidirectional Encoder Representations from Transformers.

^b^ITPT: intermediate-task pretraining.

**Table 6:** Results (counts) of error analysis for false negative errors for the Observation entity type.

Error type	Stanza [48], n	ClinicalBERT^a^ with ITPT^b^, n
Gold standard	4	4
Nonobservation	14	15
Mental or substance	13	6
True	19	25

^a^BERT: Bidirectional Encoder Representations from Transformers.

^b^ITPT: intermediate-task pretraining.

The BERT-based model is more likely to correctly annotate spans of medical procedures or traumatic injuries. This may be a consequence of fine-tuning. Interestingly, these entities are identified inconsistently throughout the data set; that is, examples of this class can be found both in FPs (“These last two maternal aunts have had hysterectomies” where hysterectomies is an FP) and FNs (“The patient's maternal grandmother died at 83 of diabetes and asthma and had a broken hip,” where *the broken hip* is an FN, undetected by the system).

Both models produce a similar proportion of errors resulting from annotating negated or general contexts (not pertaining to a specific FM). For both models, spans of this type appear among FPs (“There is no known history of ADHD or schizophrenia,” attention-deficit hyperactivity disorder [ADHD] and schizophrenia are the erroneous predictions of the systems) and FNs (“Overall, the family history is not significant for mental retardation, birth defects, multiple miscarriages or neonatal death, or known genetic conditions”; “genetic conditions” is present in the gold standard but missed by the systems).

Finally, the BERT-based model makes some mistakes by selecting spans that do not correspond to valid observations. This might be because of the model being fine-tuned on a small amount of noisy data (examples of negated contexts and traumas/procedures are mentioned earlier).

The analysis of FNs for both models shows a similar trend regarding true errors. That is, a large proportion of the observations missed by both models corresponds to expressions such as “They do not look different from other members of the family, and do not have any major internal birth defects,” where the missed span appears in a negative context. Interestingly, this is also true for the off-the-shelf model, which may suggest an inherent problem with negations (as the noisy fine-tuning data cannot be blamed with the off-the-shelf model, which does not undergo fine-tuning altogether).

Similarly, both models struggle with detecting questionable observations. For example, “She attended elementary school and could not walk until the age of 0,” where “could not walk” is the gold standard annotation.

A key noticeable difference between the models is that the fine-tuned ClinicalBERT with ITPT does a better job at detecting mental disorders and behavioral traits (eg, “Maternal grandfather, age 67, smokes but is healthy”), resulting in a 50% decrease in this type of errors. This suggests that fine-tuning is beneficial.

#### Learning Points From Annotation of Observations

The first learning point from our experiments is that an off-the-shelf state-of-the-art model trained for annotating diseases on the NCBI-disease data set provides a strong baseline, which yields much fewer true FP errors than the precision alone suggests. The analysis of the FPs shows that most of the predictions from the models are actually correct in the sense that they correctly identify Observation candidates. It is the detection of only those mentions that are linked only to specific FMs that are the most problematic. This type of constraint is inherently application specific (eg, if the aim were to assess the genetic risk of the child from FH notes collected during pregnancy, then Observations from the patient's partner FH would also be relevant). This means that an off-the-shelf model may be a good starting point for some applications.

Second, we have demonstrated a cumulative value of DAPT and ITPT. This finding highlights an important advantage of the BERT-CRF-based architecture over other state-of-the-art NER approaches, such as BiLSTM. BERT-based architectures offer an out-of-the-box transfer learning framework, with a focus on sharing models domain adapted on huge corpora (eg, BioBERT and ClinicalBERT used here).

We have also identified the key improvements achieved via fine-tuning by exposure to actual in-domain training data. The fine-tuned model provides better recall, at the expense of precision, achieving the highest aggregated F1 score (although the best-case scenario, ie, the best of our 5 models, outperforms the baseline both in terms of recall and precision). Fine-tuning contributes to better adjustment to the specificity of the task, such as tying Observations to particular FMs. More importantly, however, the model learns to detect behavioral and mental health issues from limited training data, thereby providing a qualitative improvement. This improvement, in this particular evaluation scenario, outweighs the downside of tuning the model on a relatively noisy and low-volume sample of in-domain data (which may result in some loss of precision).

Finally, we observe that, even in the fine-tuned model, there is still room for improvement with respect to adherence to the restrictions relating to the interplay between the observations and FMs. This means that we do not fully capitalize on BERT's capability to capture long-distance relationships in text. In fact, in our experiments, raw BERT-CRF yields a similar F1 score to that of the long short-term memory–based off-the-shelf baseline. We hypothesized that using an alternative training approach or using a network that jointly models both entity types could be the key to better alignment to this specific task.

### Annotation of FMs

#### Impact of Different System Elements

To provide more insight into the effectiveness of our rule-based approach in FM detection, we analyze the errors generated by our best system in row 1 of Table 4.

An ablation study, where we disable one rule at a time, is presented in Table 2. It can be immediately noted that R6 (cousin rule 2; row 10 of Table 4) is a nonfactor. Indeed, it only changes the default behavior (of adding one annotation per surface form, without changing the relationship type), when *cousin* annotation is affected by a candidate mention of any of the grandparents (eg, “grandmother's cousin”). In such cases, the *cousin* mention would not be added to the output. The results show that such an interaction between mentions is not detected in either of the training or test data sets.

In addition, removal of R3 (grandparents rule; row 7 of Table 4) and R5 (cousin rule 1; row 9 of Table 4) minimally increases the effectiveness of our system on the training data set, which does not hold for the evaluation of the test set. Elimination of all other rules (R1, R2, and R4 corresponding to rows 5, 6, and 8, respectively, of Table 4) from the best system impacts the results negatively consistently across data sets.

BERT-based parameter filtering (absent in row 4 of Table 4) impact in the test set evaluation can be seen as a sanity check. As the model was trained on the entire training data set, we assume that it can determine which paragraphs should yield no annotations, as this data set was seen at the training time. Therefore, recall is almost unaffected (we use a cutoff threshold lower than 0.5, which explains the minor change), whereas precision improves, as no annotations are generated from the paragraphs that contain no gold standard annotations. In the test set evaluation, we can see that the BERT-based paragraph filter increases effectiveness in terms of F1 score, but there is some precision-recall trade-off. The increase in precision of approximately 3.5% points comes at a cost of approximately 1% decrease in recall. This decrease in recall can be attributed to 2 types of situations:

- Paragraphs are filtered out correctly but contribute to an annotation missed elsewhere in the text.
- Paragraphs are incorrectly filtered out (misclassified).

By comparing rows 1 and 2 of Table 4, we can see that restriction on the relationship set works in the same direction as BERT-based filtering, which uses only specific relations to increase precision at the expense of recall. Results indicate that it yields a better F1 score; therefore, in terms of F1 optimization, the gain in precision outweighs the loss in recall.

Our experiments with coreference resolution, row 3 of Table 4, show that applying conference resolution within a rule-based system does not improve the annotation effectiveness. Error analysis indicates that coreference resolution often gets it wrong in grammatically ambiguous cases, such as “The patient's mother is 61 and well. Her brother, aged 21, is healthy....” The coreference module, which is trained in isolation from the task, resolves pronouns in a strictly language-focused manner. Resolving the *her* pronoun as a reference to the patient's mother (and consequently producing an *Uncle* annotation) is grammatically plausible but is unlikely from the annotation standpoint. The results and subsequent analysis indicate that the use of coreference leads to an accumulation of such cases, thereby reducing annotation effectiveness.

The results do not point to the standout importance of a single specific technique of those included in our best-performing system. Nonetheless, a combination of the rules with BERT-based filtering and a refined subset of family relationships improves the test F1 score by almost 6% points. We believe that this finding points to the accumulative potential of small improvements in rule-based systems.

#### Comparisons With the BERT-CRF Baseline

Although our best system (row 1) and other variants yielding similar performance outperform the BERT-CRF baselines (rows 12-14) in absolute values over all metrics, these differences are not statistically significant. Our initial assumption was that the N2C2 FH extraction training data set is too small to successfully train a fully ML-based model for this problem. This assumption ultimately led us to develop a hybrid solution to the FamilyMember annotation problem.

The lack of statistical significance in the advantage of our hybrid model against a strong neural (state-of-the-art) baseline suggests that the assumption was not entirely valid. In fact, the relatively strong performance of the BERT-CRF baseline indicates that this model can cope with the FamilyMember annotation and normalization, despite the small size of the training data set. However, this result also suggests that our hybrid model yields results comparable with those of a state-of-the-art ML model. It is worth noting that a purely rule-based version of our system (without ML components whatsoever; row 4) still yields comparable results, which would make it an effective, simple baseline (without the need for retraining any of the system's elements) to be considered as a starting point reference for real-world deployment of FH annotation systems. Nevertheless, the impact of individual rules still needs to be considered in the context of the target corpus and task.

Finally, our hybrid system allows for relatively intuitive prioritizing of specific performance aspects (eg, prioritizing recall over precision) by tuning system settings (row 2 of Table 2).

#### Sentence-Level Error Analysis: FP

We present the findings of a full error analysis performed on the results of our best-performing system (row 1 of Table 2) on the test data. To classify the errors, we examine individual instances in which sentence-level annotations contributed to incorrect predictions. The percentages correspond to sentence-level observations. For example, in a hypothetical passage “Mrs. X has one child, a healthy son. She also has a healthy brother whose partner recently gave birth to a daughter, and a sister, who also gave birth to a healthy daughter,” the incorrect annotation *daughter* counts twice (once per occurrence).

We categorize the FPs into the following classes: nonerrors (annotations that we believe to be correct but are not present in the gold standard), nonmentions (relationship word is used but does not denote this particular FM), partner's family (annotations pertinent to the family of the partner rather than the patient), deficiency of rules (when an expression is worded in a way that the rules miss it altogether or produce an undesired output), lack of coreference (context from outside the sentence is missing to produce a correct annotation), wrong family side (maternal/paternal heuristic failing), and other (the annotation looks fine, but even after reading the entire note, we were not able to tell if it is an actual nonerror). We analyze all errors detected on the test set and provide counts for each of the classes together with examples (Table 7).

**Table 7:** Error classes for false positives with counts and examples obtained for the best-performing FamilyMember extraction.

Class	Count, n	Example	Prediction
Partner’s family	39	“Mr William’s [from context: Mr Williams is the husband of the patient—Ms Williams] father has a brother who is currently healthy”	(Uncle, paternal)
Nonmention	38	“States on her father's side, ‘there is untreated depression’”	(Father, N/A^a^)
No coreference	32	“She [sister] has a 2-year-old son”	(Son, N/A)
Nonerror	31	“Mrs Alexander's paternal grandmother reportedly had one miscarriage”	(Grandmother, paternal)
Rules	25	“Mrs William has a healthy 30-year-old sister who has a healthy son and a daughter who...”	(Daughter, N/A)
Other	10	“Noah's mother died at age 72”	(Mother, N/A)
Wrong side	2	“...maternal paternal cousin...”	(Cousin, paternal)

^a^N/A: not applicable.

Partner's family annotations constitute the largest group of errors (approximately 23%), despite the use of the BERT-based paragraph filter. Without the filter, the number increased by more than 100%.

A large proportion (approximately 38%) of the errors fall into nonmention (approximately 21%) and nonerror (approximately 17%) categories. The distinction between these 2 classes is not always easy; for example, we classify an annotation of father in “[Patient's] father works in landscapin.” as a nonmention, although it could well be interpreted as a nonerror. We believe this explains some of the differences in precision between the test set and the training set. On the training set, these 2 classes of FPs account for approximately 30% of total errors.

Lack of coreference contributes to approximately 18% of the errors. A closer look into the problem shows that many of these errors would require long-distance contexts (more than one sentence) to correctly resolve the references. A fairly common pattern is: “Patient's father suffers from.... His brother.... A sister....” The reference (brother/uncle) from the middle sentence can be resolved correctly fairly easily using a coreference resolution module. The reference form of the last sentence, however, is not explicit, and it requires context from both previous sentences. This points to an inherent limitation of our approach of applying the coreference resolution in the scope of sentence pairs.

Errors related to rule deficiencies account for approximately 14% of all FPs. The majority of these errors are related to the fact that our approach does not deal with enumerations, as only adjacent candidates are considered in rule-based processing (as shown in the example in Table 7). This problem can potentially be solved by incorporating sentence syntactic parsing. However, sentence parsing could introduce another algorithmic source of errors because errors in parsing caused by, for example, punctuation errors that are common in medical notes propagate into the downstream task.

Other errors refer to cases in which we were unable to determine whether the annotation is a nonerror or not. These notes refer to many different people by their first names, without explicitly stating who is the main subject of the note. A context external to the note might be necessary to produce the correct annotations.

Finally, sentence-level analysis shows that the family side heuristic works exceptionally well, producing very few errors on rare occasions, such as with double cousins, as shown in Table 7.

#### A Closer Look at Coreference Resolution

To provide a better insight into the difficulty of incorporating a coreference resolution into a rule-based system, we compare the sentence-level analysis presented earlier with a similar experiment performed with the coreference resolution module.

We observe that the total number of errors is only 13 at the sentence level, but different errors are made. It is the larger variety of errors that contributes to lower precision. To provide an example of a common pattern, we can consider the following passage: “Her mother is healthy at age 63. Her father died at age 48 of COPD (Chronic Obstructive Pulmonary Disorder).” A system without the coreference resolution will produce correct *Mother* and *Father* annotations. A system with coreference resolution produces *Mother* and *Grandfather, Maternal* annotations, the latter being incorrect. Although it is incorrect, it is plausible both context-wise and grammatically. It is the accumulation of this type of mistake that negatively affects the precision score of the system with coreference resolution. We believe that a key takeaway is that in ambiguous cases (without explicit specification), choosing the patient as a reference point for a family relation is statistically safer than the coreference approach.

#### Missed FamilyMembers: FNs

Analyzing FNs within the test set is an inherently labor-intensive task, as it requires inspecting the entire FH note (to find the sentence-level evidence and identify why the system got it wrong). Our selective analysis indicates that a large proportion of the annotations missed by our system are related to the nonerrors detected in the exploration of FPs. For example, for the passage,

“This maternal aunt has three healthy children, but also had a daughter that died within the first few days of life secondary to hydrocephaly,” our system provides an annotation (Cousin, Maternal). The gold standard requires an annotation of (Cousin, NA), which we assume relates to this particular text. As we believe the output of our system is correct, we classify it as a nonerror. At the same time, by correctly interpreting the sentence-level evidence, the system misses the gold standard annotation and the same nonerror penalizes both recall and precision.

#### Learning Points From FamilyMember Annotation

Our experiments with the FamilyMember annotation point to several high-level conclusions, which may be relevant for future work in this domain. First, the careful optimization of the system (error analysis on training data for debugging, choosing a more reliable set of relationships, and introducing BERT-based filtering) improves the overall performance of the system by more than 6% points, which we consider to be a fairly encouraging result. We are convinced that these results can be pushed even further with minor tweaks; however, it would be difficult to point to a specific thing that would drastically improve effectiveness if fixed. In addition, crafting additional rules that are very specific to the relatively few observed errors carry a risk of overfitting. In this sense, our approach has been taken relatively far for effective tuning.

Second, our experiments with coreference resolution demonstrate the intrinsic difficulty of configuring a *language understanding* component as an add-on to a rule-based system. We imagine that it is possible to come up with a much broader rule set that could take advantage of the coreference resolution. Nonetheless, not all context understanding can be solved with coreference resolution, especially for grammatically ambiguous cases or when deciding whether a matching surface form is an actual mention or a nonmention. This is even less useful when the coreference resolution is trained without task-specific context understanding. We believe that this points to a general limitation in rule-based approaches to the problem.

The use of pretrained neural LMs is the most viable path toward incorporating language understanding in the FamilyMember annotation. In our experiments, we demonstrate a simple BERT-based paragraph filtering approach, which improves the effectiveness of the final system. Its incorporation is easier than that of coreference resolution because we identify an isolated task (the interaction with the rest of the system is simple), which has task-specific training data (the method sees the contexts from a task-specific perspective). Nevertheless, a fully optimized ML baseline (BERT-CRF) does not outperform the rule-based approach.

In an ideal scenario, with thousands of training records, the FamilyMember annotation problem could be approached identically to that of Observations. However, with limited training data available for the task, such a model achieves effectiveness slightly lower than that of a rule-based system, as per our baseline experiments.

The third important takeaway relates to another advantage of a rule-based system, beyond the possibility of tuning F1 with very little training data. The rule-based system can generate conceptually correct annotations, regardless of the quality or completeness of the training data. We believe this is the reason we see so many nonerror FPs—our rules are conceptually sound. Therefore, the system will generate those outputs, even if the training data often miss a particular type of relationship. This means that rule-based systems, or their combinations, play an important role in creating annotated data sets that are needed to train deep learning approaches.

### Overall Effectiveness of the System

We provide an overview of our best system’s performance for FamilyMember and Observation annotations combined, compared with other approaches on the same data set, as shown in Table 8. As the FH notes collection is relatively new, most systems we compare against are those that participated in the 2019 N2C2 shared task (we selected the top 5 runs). We are aware that this comparison is not entirely fair, as we continued refining our system after the release of the test data, but it does put our results in perspective. For our best run, we present a combination of DAPT (ClinicalBERT) with ITPT (NCBI-disease) for Observation annotation and the best-performing system for the FamilyMember annotation. We train the neural component for Observations with 5 different seeds and report average results with SDs.

The combined results demonstrate that the proposed system performs on par with top systems from the 2019 N2C2 shared task, with the exception of the Harbin Institute of Technology (HIT) team’s approach, which achieves superior precision. We believe this is because of HIT proposing a model that jointly addresses both FamilyMember and Observation mentions via ML. It seems that their approach aligns better with the perks of this specific task (eg, annotating only diseases pertinent to specific FamilyMembers). In addition, our experiments with the BERT-CRF baseline for FamilyMember annotation indicate that the gap cannot be easily closed by simply using a state-of-the-art NER model for FamilyMember annotation. This also indicates that the key source of the difference in effectiveness between our best system and that of HIT is the HIT's feature of joint modeling of FamilyMember and Observation mentions.

**Table 8:** Comparison with other systems for both types of mentions combined^a^.

Run	Precision	Recall	F1 score
Our best run, mean (SD)	79.60 (2.2)	83.64 (1.2)	81.63 (0.8)
HIT^b^	91.54	83.72	87.45
EZDI	80.90	83.65	82.25
MUSC^c^	78.90	83.84	81.30
NTTU^d^	80.43	80.93	80.68
UF^e^	79.69	79.20	79.44
N2C2^f^ official median	—^g^	—	76.59
A1^h^ [28]	65.01	88.92	75.10
A2^h^ [28]	85.07	62.11	71.80

^a^National Natural Language Processing Clinical Challenges median is calculated from all valid runs participating in the original evaluation within the shared task.

^b^HIT: Harbin Institute of Technology.

^c^MUSC: Medical University of South Carolina.

^d^NTTU: National Taitung university.

^e^UF: University of Florida.

^f^N2C2: National Natural Language Processing Clinical Challenges.

^g^Not available.

^h^These are variants of the system described in the cited study.

In this study, we investigate the impact of a set of techniques (DATP and ITPT for disease annotation; rules and paragraph filtering for annotation of FMs) to improve the performance of a very simple yet reasonably effective baseline system (78.10 and 75.86 F1 scores for Observations and FamilyMembers, respectively, place it close to N2C2 median performance). Our experiments suggest that the proposed improvements, although subtle, generate a considerable cumulative effect, resulting in a final system performing at a close to state-of-the-art level. We also present a detailed error analysis for errors for the relatively less explored problem of annotating FMs in clinical notes.

### Conclusions

We investigate the problem of detecting diseases and FM mentions in FH reports. We propose an approach that leverages state-of-the-art NER for disease mention detection, coupled with a hybrid method for FM mention detection. The hybrid method implements a rule-based approach combined with a text classifier to filter out irrelevant paragraphs from the reports (eg, pertaining to the patient partner's family).

Our approach achieved effectiveness close to the top 3 systems participating in the 2019 N2C2 FH extraction challenge, with only the top system outperforming it convincingly in terms of precision.

We believe that immediate improvements could be achieved by refining the rules used in the FM mention detection module. Nonetheless, alternative strategies, revolving around the use of semisuperised and distantly supervised learning, are closer to our research interests. A more encompassing approach toward improving performance would be a system that jointly models diseases and FMs, thereby improving cases that relate directly to the interplay between both entity types (eg, not annotating diseases of nonblood-related FMs).

In our future work, we will concentrate on applying FH extraction to a broader set of medical notes. This broader approach will not only cater to new use cases but will also allow for harnessing the FH-related knowledge scattered across other sections of EHRs.

## Appendix

Multimedia Appendix 1 Implementation details. The configuration of our BERT model follows the original BERT-based model. In particular, our model is based on a bidirectional transformer with 768 hidden dimensions, 12 hidden layers, and 12 self-attention heads. The total number of parameters was approximately 110 million. We implemented our model using PyTorch and trained it using 1 RTX 2080 Ti graphics processing unit. As the training set size is small, iterating all instances once (1 epoch) takes less than 15 seconds. We adapted the early stop method, wherein the training will stop once there is no improvement (measured on the development set) during the last 5 consecutive epochs. The trained model that was most effective on the development set (measured using the F1 score) was used to evaluate the test set. BERT: Bidirectional Encoder Representations from Transformers.

## Abbreviations

BERT: Bidirectional Encoder Representations from Transformers
BiLSTM: bidirectional long short-term memory
CRF: Conditional Random Field
CSIRO: Commonwealth Science and Industrial Research Organization
DAPT: domain-adaptive pretraining
EHR: electronic health record
FH: family history
FM: family member
FN: false negative
FP: false positive
HIT: Harbin Institute of Technology
i2b2: Integrating Biology and the Bedside
IE: information extraction
ITPT: intermediate-task pretraining
LM: language model
ML: machine learning
N2C2: National Natural Language Processing Clinical Challenges
NCBI: National Center for Biotechnology Information
NER: named entity recognition
NLP: natural language processing
REX: Regenstrief Data Extraction
ShARe-CLEF: Shared Annotated Resources-Conference and Labs of the Evaluation Forum
TP: true positive
