# Correction: Extracting Family History Information From Electronic Health Records: Natural Language Processing Analysis

**DOI:** 10.2196/30153

**Published:** 2021-05-03

**Authors:** Maciej Rybinski, Xiang Dai, Sonit Singh, Sarvnaz Karimi, Anthony Nguyen

**Affiliations:** 1 Commonwealth Scientific and Industrial Research Organisation Sydney Australia; 2 University of Sydney Sydney Australia; 3 Macquarie University Sydney Australia; 4 Commonwealth Scientific and Industrial Research Organisation Brisbane Australia

In “Extracting Family History Information From Electronic Health Records: Natural Language Processing Analysis” (JMIR Med Inform 2021;9(4):e24020) one correction was made.

Due to a system error, five extraneous figures were added to the Methods section of the paper at the time of publication. These have been removed from the corrected version.

The correction will appear in the online version of the paper on the JMIR Publications website on May 3, 2021, together with the publication of this correction notice. Because this was made after submission to PubMed, PubMed Central, and other full-text repositories, the corrected article has also been resubmitted to those repositories.

