# Supplementary material for: Extracting Family History Information From Electronic Health Records: Natural Language Processing Analysis
Source: JMIR Med Inform. 2021 Apr 30;9(4):e24020. doi: 10.2196/24020 (PMC8092929; doi:10.2196/24020)
Supplement: Multimedia Appendix 1 [file medinform_v9i4e24020_app1.docx]

## Appendix 1: Implementation Details

The configuration of our BERT model follows the original BERT base model. In particular, our model is based on the bidirectional transformer with 768 hidden dimensions, 12 hidden layers, and 12 self-attention heads. The total number of parameters is around 110M. We implement our model using PyTorch and train it using one RTX 2080 Ti GPU. Because the training set size is small, iterating all instances once (one epoch) takes less than 15 seconds. We adapt early-stop where the training will stop once there is no improvement, measured on the development set, during the last five consecutive epochs.

The trained model which is most effective on the development set, measured using the F1 score, is used to evaluate the test set.
